# Supplementary material for: Social-ecological alignment and ecological conditions in coral reefs
Source: Nat Commun. 2019 May 3;10:2039. doi: 10.1038/s41467-019-09994-1 (PMC6499785; doi:10.1038/s41467-019-09994-1)
Supplement: Supplementary file 1 — Supplementary Information [file 41467_2019_9994_MOESM1_ESM.pdf]

Supplementary Information file

Barnes et al. 2019

Social-ecological alignment and ecological conditions in coral reefs

Supplementary Methods

We collected detailed social network and sociodemographic data from ≥ 75% of the estimated population of fishers across five coral reef fishing communities along the Kenyan coast from December 2015 to May 2016 (Supplementary Figure 1). The sample statistics and social network metrics are reported in Supplementary Table 1, actor attributes are reported in Supplementary Table 2, and the social networks are depicted in Supplementary Figure 2.

The network boundary was defined as fishers operating within each community. Accurate estimates of total fisher populations in different communities along the Kenyan coast are difficult to obtain. However, we avoided sites where fishing populations were assumed to be >300, as this would have made data collection for a complete network design untenable. Estimates of total fisher populations were updated upon arrival in each site in consultation with formal community leaders prior to data collection. In our name generator used to collect the social network data, we asked for full names and included name qualifiers (e.g., where does this person live/work?) to ensure identification accuracy of all nominated individuals. Names and qualifiers were checked daily while in the field and against all previously collected data, and all individuals (both respondents and nominated alters) were assigned a unique ID. Following a complete network design, our analysis focused on ties among respondents only; thus, ties to non-respondent fishers and other actors outside our population of interest (e.g., NGO representatives, government officials, etc.) were dropped.

The fishing communities we focused our analyses on are representative of a social community of fishers, each with associated fishing areas adjacent to their community that they use and have rights to manage[1]. It is important to note that this is not synonymous with fish landing sites, i.e., some landing sites are frequented by fishers from neighboring communities (Supplementary Table 2). A minority of fishers we interviewed (n=45 out of 648) associated themselves with, and fished in, two of the communities we studied: sites A and D, which are in close proximity to each other (4.5 km apart). These 45 fishers were therefore included in our network analysis of both sites, which is reflective of their social life and fishing behavior. To investigate if our analysis was sensitive to these 45 fishers accessing both sites, we ran our network models for sites A and D both with and without these individuals, and found no meaningful difference in our results (further details regarding model fit, residual effects, and our sensitivity analyses are included on pages 5-8 of this document).

The ecological network captures trophic interactions (i.e., predator-prey relationships) among target reef fish comprising the majority of catch by all fishing gears employed in our five study sites. To determine which species were caught by each gear type being used by fishers across our study sites, we used a long-term fish catch dataset collected by our partners at the Wildlife Conservation Society. The dataset included surveys from 25 landing sites along the Kenyan coast conducted continuously between 2010 and 2016. For each observation, onsite observers identified landed catch at the species level in addition to the gear used. Observers were present at landing stations every sampling day before the arrival of boats and stayed until the entire landing process was concluded. Although all patrols were conducted during daylight hours, the sampling method does not exclude catches attributed to nighttime fishing activities, as observers also intercepted fishers returning from their overnight fishing, ensuring that each gear used at each site was sampled and that each species landed was recorded. The number of patrols conducted per month were not stratified, but similar intervals of sampling were maintained within this randomized block design to detect long-term catch trends. Data was

collected at least 8 days per month, translating to a total of 599 sampling days over the survey period and an observed total of 19,467 individual fish caught across all gear types. Most gears used in multispecies coral reef fisheries incidentally catch a number of species infrequently. We therefore focused on species comprising the majority of the total catch for each gear type, excluding all species that comprised less than 1% of the total catch (Supplementary Table 3).

Trophic interactions capturing predator-prey relationships among the 36 target reef fish species were estimated based on a combination of diet, relative body size, and habitat use (likelihood of encounter). The corresponding ecological network was thus undirected, with edges representing trophic interactions between fish species B_u and B_v (Supplementary Figure 3). Diet and body size (maximum length) data were taken from *FishBase*[2], and broad patterns of habitat use (i.e., pelagic, demersal, coral-dominated, macroalgal bed) were estimated from published records and expert first-hand knowledge (TRM, NAJG, ASH). Detail on the specific fish species consumed from analyses of gut contents is largely unavailable, yet piscivorous coral reef fish are known to be generalists in terms of the species they consume[3]. We thus took a conservative approach, considering one species to prey on another if its diet was predominantly piscivorous, its body length was large (ca. ≥ 2 times) compared to that of the prey species, and the two species occupied a similar habitat. We did not identify potential predatory links for any species whose diet was not primarily piscivorous as any likely predation on other targeted species was likely to be too infrequent to have a meaningful effect on prey populations. The resulting network included 36 nodes, 244 ties, a mean geodesic distance of 1.919, an average degree of 6.778, and one component (Supplementary Figure 3). The trophic matrix is available at http://dx.doi.org/10.25903/5c89d99f5d654.

Though switching fishing gears throughout the year depending on the season is quite common in many fisheries around the world, the majority of reef fishers in our study sites used only one primary gear type year-round. Social-ecological ties were therefore identified by linking individual fish species to individual fishers via their primary fishing gear as identified by fishers in our fisher survey (Supplementary Table 4). In other words, if fisher A_i used gear type G_t as their primary gear, and gear type G_t targeted fish species B_u, a social-ecological link would exist between fisher A_i and fish species B_u.

ERGMs are statistical models of networks based on explicit hypotheses about network dependence[4]. Treating each network tie as a random variable, ERGMs model network ties explicitly, specifying the probability of observing the network (Y) with n nodes as a function of local network processes in the following form:

$$\Pr(Y = y) = \frac{1}{\kappa(\theta)} exp \sum_Q \theta_Q z_Q(y) \quad (1)$$

where y is a network instance; Q defines particular network configurations which are based on tie dependence assumptions that capture social processes; $z_Q(y) = \sum_y \prod_{Y_{ij} \in Q} y_{ij}$ is the network statistic for the corresponding network configuration of type Q, θ_Q is the parameter associated with $z_Q(y)$; and $\kappa(\theta)$ is a normalizing constant defined based on the graph space of networks of size n and the actual model specification. Note that a network variable Y can be seen as a collection of tie variables (Y_{ij}) defined on each dyad (i, j) of the network, whereas as network configuration of type Q includes tie variables that are conditionally dependent given the rest of the network.

Multilevel ERGMs can be seen as an extension of ERGMs that account for networks linked across multiple levels[5]. Here, network ties are considered interdependent not only within levels but also across levels, enabling the interpretation of cross-level interactions and configurations (e.g., Fig. 1). Multi-level ERGMs for two-level networks, such as the social-ecological networks studied here, can be expressed as:

$$\Pr(A = a, X = x, B = b) = \frac{1}{\kappa(\theta)} exp \sum_{Q} \{z_Q(a) + \theta_Q z_Q(x) + \theta_Q z_Q(b) + \theta_Q z_Q(a,x) + \theta_Q z_Q(b,x) + \theta_Q z_Q(a,x,b)\} \quad (2)$$

where $z_Q(a)$ and $z_Q(b)$ are network statistics for the within level network configurations (A and B, Fig. 1); $z_Q(x)$ are network statistics for structural effects within the bipartite affiliation network (X, Fig. 1); $z_Q(a,x)$ and $z_Q(b,x)$ are network statistics for configurations involving ties from one of the unipartite networks (A or B) and the bipartite network (X), thus representing interactions between the two networks, such as the social-ecological triangle of primary interest here (Fig. 1); and $z_Q(a,x,b) = \sum_{a,x,b} \prod_{A_{ij} \in Q, X_{kl} \in Q, B_{uv} \in Q} a_{ij} x_{kl} b_{uv}$ are statistics for configurations involving ties from all three networks.

Building on social selection models[6], an extended version of multilevel ERGMs incorporate nodal attributes as exogenous covariates in order to account for their ability to effect network structures. This extension, described in detail in Wang et al. [7], can be expressed as:

$$\Pr(A = a, X = x, B = b \mid Y^A = y^A, Y^B = y^B) = \frac{1}{\kappa(\theta)} exp \sum_{Q,\Lambda} \{ \theta_Q z_Q(a,x,b) + \theta_\Lambda z_\Lambda(a,x,b,y^A,y^B)\} \quad (3)$$

where $z_Q(a,x,b)$ represents graph statistics involving only network tie variables (Eq. 2); $z_\Lambda(a,x,b,y^A,y^B)$ are statistics involving interactions among tie variables and attribute values, where Λ represents configurations derived from dependence assumptions between network tie and nodal attribute variables[6]; and θ_Q and θ_Λ are the associated parameters for their corresponding graph statistics.

Here, we employed the extended version of multilevel ERGMs (Eq. 3) by testing for the closed, cross-level social-ecological triangle ('Triangle XAX', Fig. 1) in each site while controlling for well-known endogenous (i.e., structural) mechanisms involved in shaping social networks in addition to nodal attributes shown to effect information exchange ties in small-scale fisheries. To capture endogenous mechanisms, we controlled for network density, centralization, and closure in A. We included the edge parameter (EdgeA) to capture density, which corresponds to the baseline propensity for fishers to establish information exchange ties. In ERGMs, the edge parameter is akin to the intercept term in a logistic regression. We included the alternating star configuration (ASA), and in one case, the two-star (Star2) configuration, to capture centralization. Centralization is a common feature of self-organization in social networks, which captures the social process of *preferential attachment*, whereby popular actors attract even more popularity. Centralization manifests as centralized star configurations: the alternating star configuration is a weighted sum of all star counts, whereas the two-star configuration helps capture the variance in degree distribution[4]. To capture social network closure, we included the alternating triangle parameter (ATA). Social network closure (also referred to as transitivity or

clustering) captures the propensity of actors to establish ties with friends of their friends, thus forming micro-level triangles which can sometimes scale up to create subsets of actors in clique-like structures - a common feature of positive affect networks among humans[4]. The alternating triangle configuration helps to capture the idea that rather than being evenly distributed throughout the graph, triangles often tend to form together (or attach themselves to each other), forming denser regions of multiple triangulation[4].

To capture nodal attributes, we included (1) the propensity of leaders to be more active/have more ties established in the network (leader activity) and (2) the propensity of fishers using the same landing site to form ties (landing site homophily – which can be seen as a form of geographic proximity), as there is evidence that both are important drivers of positive affect social tie formation in small-scale fisheries[8]. To ensure our estimates of landing site homophily were robust, we examined the residual effects for the propensity of fishers associated with particular landing sites to establish ties (i.e., 'landing site activity') in each model following the methods laid out in [9]. Full models included controls for activity in each landing site where our residual analysis suggested fishers were more active in forming and maintaining ties than would be expected by chance alone (Supplementary Table 5). Because our focus here was on social processes - particularly the propensity for fishers to form ties if they were in direct competition over resources - the X and B level networks were fixed and treated as exogenous. Beyond the scope of this research, future work can build on this approach by testing hypotheses regarding more complex network configurations that include dependencies in the ecological network by treating the B level network as endogenous.

Complex multi-level ERGMs can suffer from convergence problems, particularly when the number of parameters initially included is high[4]. We therefore followed the conventional approach of building our models sequentially[8]. Specifically, we started with structural mechanisms in the social network, followed by nodal attribute effects, and then added the cross-level effect of interest here as the last step; ensuring convergence at each step. All models were run in MPNet[10], which implements a Markov chain Monte Carlo (MCMC) procedure to estimate model parameters using maximum likelihood estimation, as described in Snijders[11]. The dependence assumption built in to the ERGM framework makes this approach necessary, as every tie variable must be modelled conditionally dependent on all other ties in the observed network, making it unfeasible to analyse all possible graphs due to the extremely large number of possible network configurations. The MCMC routine builds a probability distribution of the model parameter estimates through random sampling in the subset of all possible networks that exhibit the structural characteristics of the observed network. This makes it possible to test whether the observed values of the parameter estimates differ significantly from what would be expected by chance alone under a random hypothesis. Full model results are presented in Supplementary Table 5.

For each final fitted model, we tested the goodness-of-fit by comparing simulated graph statistics of the estimated model with the observed network. This approach treats our final models as the null model, and analyses whether features of the simulated networks predicted by the model deviate significantly from those in the observed network. Using this approach, it is possible to examine not only fitted effects, but also the residual structural effects of graph features that were not explicitly modelled, thus providing an indication of whether the model is a good representation of how the empirical network could have been formed. The ability to analyse residuals of non-fitted effects in this way is another benefit of ERGMs relative to more traditional linear models, where interpretation is typically limited to parameters included in the

model[9]. To execute this procedure, we simulated 10 million graphs and took a sample every 10,000ths graph, resulting in a sample size of 1000. For each graph in the sample, we examined the mean and standard deviation of each graph feature and/or counts of each configuration to create distributions of graph statistics, and used the mean and standard deviation from the simulated distribution to calculate a t-ratio for each graph statistic. All of our fitted effects in each model had a t-ratio of < 0.1, confirming the estimated models had converged. Finally, although at present there is no conventional approach to compare the magnitude of cross-level effects with within-level effects in multi-level ERGMs, the Mahalanobis distance estimates (which measures how far away the observed network is from the center of the distribution of modeled networks) indicated a better model fit with the cross-level social-ecological triangle included in each model.

For non-fitted effects, we examined whether their values were extreme, i.e., if the observed data had more or less of a particular graph feature than 95% of graphs from the simulation, resulting in a t-ratio of > 2 in absolute value. We examined a total of 38-43 graph statistics, including 14 structural configurations in the social network; the global clustering coefficient and the standard deviation and skewness of the degree distribution in the social networks; 11 cross-level structural effects; and 10 to 15 nodal attribute effects, which included homophily and heterophily on ethnicity, gear type, landing site, and religion in each network, in addition to activity for each landing site represented in each network. In three of our sites, the models provide an adequate fit to all graph statistics. In sites A and C, t-ratios indicate that higher order stars capturing aspects of centralization and the skewness of the degree distribution were not well captured, despite having increased the lambda value for ATA (and for site C, ASA) to 2.5 [see Lusher et al. [4]] and fixing the tie density of one very high (i.e., extreme) degree node in each social network in our final models. We experimented with including the 2Star configuration and higher order stars to capture this, but could not get the models to converge. Degree distributions are known to be difficult to model, and as explained in [4], it is often unreasonable to expect an ERGM to fit all features of a network, just as we do not expect a regression model to explain 100% of the variance. What is important is that the model features relevant to the research question are well accounted for. As our focus here was on the closed cross-level social-ecological triangle rather than centralization in the social network, we are confident our conclusions are valid despite these features not being well represented.

As discussed previously, there was a small amount of overlap between fishers operating in site A and site D (n=45). We therefore ran our network models for sites A and D with these fishers removed following the exact procedure discussed above, and found no meaningful difference in our results. This shows that these multi-site fishers alone are not significantly different from the majority of the other fishers only operating in one of the communities/fishing grounds.

Our focus here was on the potential ecological impacts of closed, cross-level social-ecological triangles rather than their drivers (i.e., what drives fishers to form social-ecological triangles?). However, because fishing gears helped to define the social-ecological ties in the present study, gear-based effects may to some extent help to explain *why* fishers who target the same resource chose to form cooperative communication ties in some of our sites, which has important practical implications. Indeed, the value of exchanging experiences and knowledge with others using the same technology is rather intuitive – it allows actors to accrue technical knowledge that can help them to maximize harvest levels and operate more efficiently. Yet because actors using the same technology also tend to target the same resource for extraction, these exchanges can also enhance trust and a shared ecological understanding of factors

important for the resource to be sustained. As a post-hoc exploratory analysis, we therefore examined whether gear-based homophily – i.e., fishers using the same technology preferentially forming ties – may help to explain the prevalence of closed, cross-level social-ecological triangles. Clearly these two effects are difficult to disentangle because the gear fishers use will define what species they target. To explore this, we therefore removed the cross-level social-ecological triangle and instead added gear-based homophily to our models. Our results from this exploratory analysis show that gear homophily was an important driver of tie formation in sites A – C, but not in D and E. This suggests that in sites A – C, where we see significant effects of social-ecological network closure (Fig. 3), technology may act as a convening factor that brings fishers who compete over the same resources together to share knowledge and expertise. However, why technology may act as a convening factor in some sites but not others, particularly when there is little difference in the diversity of gear use across sites (Supplementary Table 2), remains an open question. Moreover, this does not negate the importance of cooperative communication ties between fishers using different gear types but targeting the same species. Indeed, many such ties are present in our empirical data, and they are undoubtedly critical for managing the destructive effects of gear competition over common species in multi-species fisheries[12].

In situ ecological conditions were examined using a combination of data and methods (Supplementary Table 7). Fish biomass (kg/ha) was measured using underwater visual census data collected across a total of 2-6 500m^2 transects in fished areas in each site from 2011-2015 (Supplementary Table 7). All sites were under 10 meters depth and most were a reef flat habitat with only one exception (site A), which was a reef slope. The abundance of all diurnally active, non-cryptic, reef-associated fishes was quantified at each site in each year, and their size (cm total length) estimated. Fishable biomass was calculated for fish >10 cm total length (TL) using known/published length-weight relationships. Smaller bodied fish (<10 cm TL) were not included because the abundance of smaller bodied fish is typically underestimated using visual census, and the fishery targets fish above this size. Of our biomass estimates, a majority is comprised of species that are shared by multiple competing fishers (e.g., 69%, 59%, 78%, 41%, and 55% of estimated fishable biomass across sites A-E in 2014 was targeted by multiple competing fishers).

The functional richness of fish assemblages from the visual surveys described above was estimated following the method detailed in [13]. In short, we assigned each species a value for six categorical traits that have been commonly used in functional diversity studies of coral reef fishes (i.e., diet, body size, mobility, time of activity, schooling behavior, and position in the water column). Each trait was comprised of several categories, resulting in 81 possible unique trait combinations. For example, based on main items consumed, diet was categorized into seven trophic categories: macroalgal herbivorous (i.e., fish eating large fleshy algae and/or seagrass), carnivorous (including fish and cephalopods), invertivorous targeting mobile invertebrate (i.e., benthic species such as crustaceans), herbivorous-detritivorous (i.e., fish feeding on turf or filamentous algae and/or undefined organic material), omnivorous (i.e., fish for which both vegetal and animal material are important in their diet), invertivorous targeting sessile invertebrates (i.e., corals, sponges, ascidians), and planktivorous (i.e., fish eating small organisms in the water column). From the traits dataset, we performed a Principal Coordinates Analysis (PCoA) to build a multidimensional functional space based on pairwise Gower's distances between species. Species coordinates on the first four principal axes (PC) of this PCoA were used to construct a synthetic multidimensional ordination from which we computed functional richness. The first four dimensions of the ordination were selected a posteriori and

single score functional richness calculated according to the position of species in this four-dimensional space. A square root correction for negative eigenvalues was applied for Euclidean representation of distance relationships among species in order to avoid biased estimations.

Biophysical variables were coral cover and rugosity. Coral cover was quantified using standard 10m long line intercept transects (Supplementary Table 7). The length of transect occupied by live corals was measured along six to nine replicate transects per site, which was converted to percent coral cover[14]. Rugosity was quantified along the same transects using the linear versus contour method, whereby the difference in distance between a tight 10m line and one that follows the contour of the reef was measured[14]. Environmental variables were sea surface temperature (SST) and net primary productivity (NPP), and human impact measures were human gravity[15], and fishing pressure. Further description of these variables, including data sources/methods, are provided in Supplementary Table 7.

As coral cover and rugosity data were unavailable for site A, we were unable to directly compare the biophysical condition of this site with the remaining four sites. As an alternative, we compared analyses of the difference in mean biomass and functional richness of fished resources between sites with and without social-ecological network closure both with and without site A using all data, and for 2014 only using one-sided t-tests and effect size estimates (Fig. 4, Supplementary Table 8). We also compared the difference in mean SST, NPP, coral cover, rugosity, human gravity, and fishing pressure between sites with and without social-ecological network closure both with and without site A for all years using two-sided t-tests and effect size estimates. As demonstrated in Supplementary Table 8, our results are insensitive to the inclusion of site A or the sampling year/s; indicating that there is no meaningful bias introduced by the inclusion of site A, or by including all available data.

Supplementary Figures

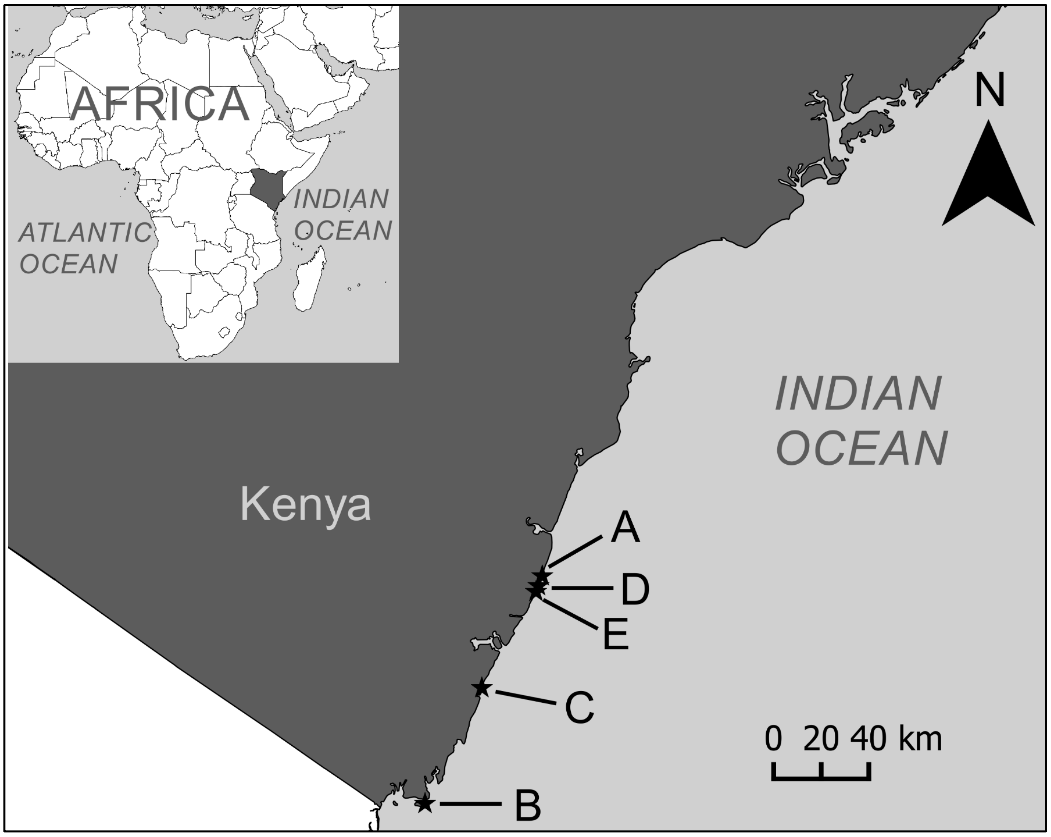

Supplementary Figure 1. Study sites. Map of the Kenyan coast showing the geographical location of the study sites. Stars indicate location of the individual sites.

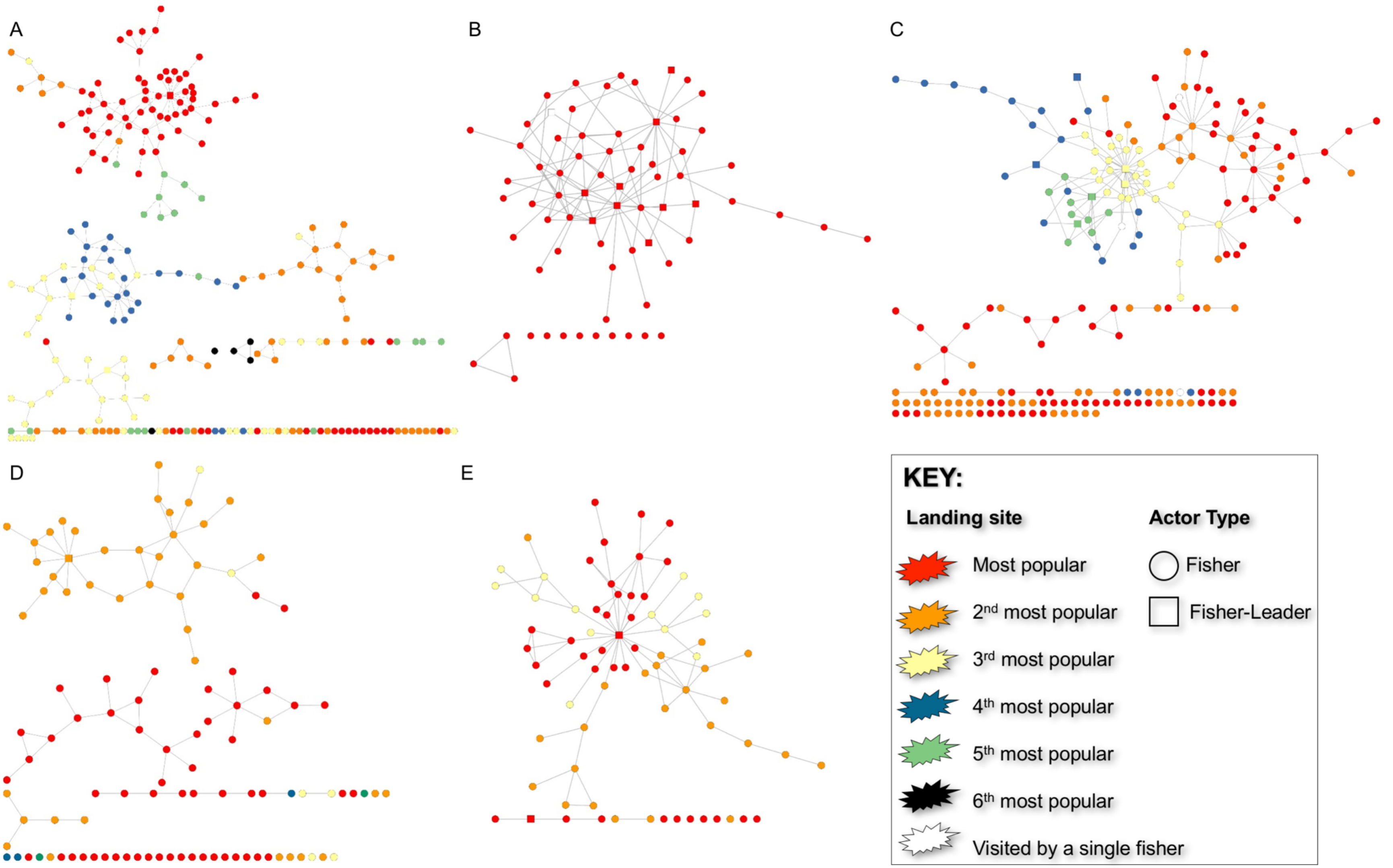

Supplementary Figure 2. Social Networks. Fishery-related information exchange among fishers across our five sites.

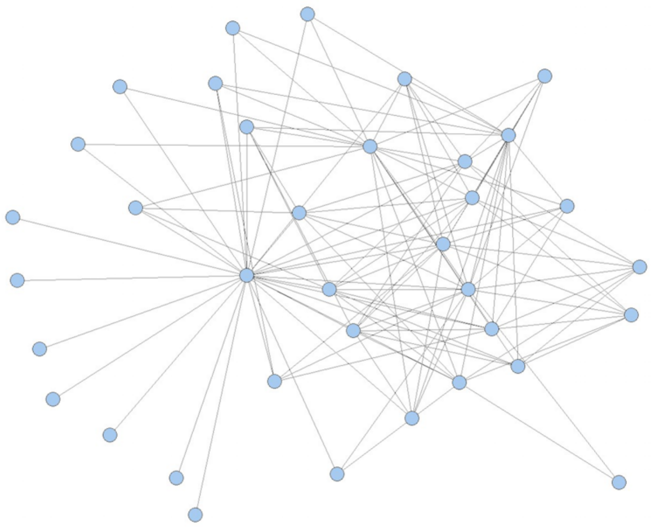

Supplementary Figure 3. Ecological network. Trophic (predator-prey) interactions among 36 reef fish comprising the majority of catch across small-scale fishing gears used in Kenya. Raw data is available at http://dx.doi.org/10.25903/5c89d99f5d654.

Supplementary Tables

Supplementary Table 1. Sample and social network metrics across sites.

	Site				
	A	B	C	D	E
Est. fisher population (N)	316	151	290	124	100
Total sample (% of est. pop)	239 (76%)	127 (84%)	218 (75%)	94 (76%)	78 (78%)
No. of individuals dropped due to gear classification[a]	11	55	10	1	4
Total remaining fishers (i.e., nodes)	232[b]	71	207	109[b]	74
Total info sharing ties (i.e., edges among fishers)	224	124	214	83	98
Connected components	15	2	13	8	3
No. of isolates	65	9	71	36	9
Max geodesic distance (diameter)	12	8	16	9	10
Avg. geodesic distance	4.46	3.09	4.70	3.74	3.76
Graph density	0.01	0.05	0.01	0.01	0.04
Avg. degree	1.93	3.49	2.07	1.52	2.65
Avg. clustering coefficient	0.30	0.30	0.33	0.24	0.39

[a] Fish catch from fishers who gleaned (e.g., 55 fishers in site B) or whose gear type was identified as “other” was not available. Thus, actors whose only method of fishing was gleaning and/or listed as “other” were not included in this analysis.

[b] Sites A & D are located in close proximity to each other, and a minority of fishers overlap between these two sites (n = 45). Some respondents from site A who did not associate themselves with, or fish in, site D were nonetheless identified as important for information exchange by fishers in site D. They were therefore included in site D’s network.

Supplementary Table 2. Actor attributes by site.

	Site				
	A	B	C	D	E
<u>Gear type</u>					
Line	48 (21%)	49 (69%)	86 (42%)	28 (26%)	16 (22%)
Gillnet	100 (43%)	2 (3%)	25 (12%)	36 (33%)	39 (53%)
Seine	3 (1%)		1 (1%)		1 (1%)
Spear	81 (35%)		55 (27%)	45 (41%)	18 (24%)
Trap		20 (28%)	40 (19%)		
<u>Landing site</u>					
1		70 (99%)	10 (5%)		
2	20 (9%)			56 (51%)	
3					
4			64 (31%)		
5	47 (20%)			2 (2%)	
6			27 (13%)		
7	5 (2%)				
8					13 (18%)
9			1 (<1%)		
10	54 (23%)			6 (6%)	
11					
12		1 (1%)			
13					37 (50%)
14			81 (39%)		
15	27 (12%)			3 (3%)	
16					24 (33%)
17			1 (<1%)		
18			22 (11%)		
19	79 (34%)			42 (39%)	
20			1 (<1%)		

Supplementary Table 3. Species caught by gear type. Values equal the percent of each species comprising the total catch per gear type. Species are listed in order of the most predominately caught across all gears.

Species	Spear gun	Hook & line	Gillnet	Seine nets	Traps
Siganus sutor	8.2	3.5	14.6	16.9	40.6
Leptoscarus vaigiensis	32.9		5.2	12.7	5.6
Lethrinus lentjan		11.6	4	2.9	3.8
Lethrinus harak		7.5	10.9	4.3	
Lutjanus fulviflamma	2.8	11.8	4.1	3.3	1.3
Lethrinus mahsena		15.2	2.5	4.1	2.7
Calotomus carolinus	5.8		5	3.2	2.3
Acanthurus triostegus	3.60		7.3		2.0
Parupeneus macronemus	2.9	2.6	2.6		1.3
Ctenochaetus striatus	4.8		3.3	3.2	
Cheilio inermis	2.2	5.2	2.8	3.8	
Parupeneus barberinus			1.7	3.9	4.2
Hemiramphus far			1.7	1.9	
Cheilinus trilobatus	2	4.7			
Strongylura incisa				1.2	
Lethrinus olivaceus		6.1			
Sphyraena barracuda			2.6	2.5	
Sardinella gibbosa				1.5	
Sphyraena flavicauda				4.3	
Lethrinus nebulosus		2.9			1.3
Pempheris adusta					
Gerres oyena			1.7		
Scarus ghobban					3.3
Thalassoma hebraicum		3.1			
Plectorhinchus flavomaculatus	2.8				
Parupeneus indicus					2.5
Scarus atrilunula				2.0	
Scomberoides commersonianus			1.9		
Plectorhinchus gaterinus				1.8	
Rastrelliger kanagurta				1.8	
Lutjanus bohar		1.7			
Halichoeres hortulanus		1.5			
Siganus stellatus					1.5
Acanthurus nigrofuscus					1.4
Acanthurus tennenti					1.3
Abudefduf vaigiensis				1.2	
Zanclus cornutus					1.2

Supplementary Table 4. Target fish species. Number and percentage of total fishers in each site targeting each species. Species are listed in order of most predominantly targeted across all sites.

	Site				
Fish species	A	B	C	D	E
Lutjanus fulviflamma	232 (100%)	71 (100%)	207 (100%)	109 (100%)	74 (100%)
Siganus sutor	232 (100%)	71 (100%)	207 (100%)	109 (100%)	74 (100%)
Parupeneus macronemus	229 (99%)	71 (100%)	206 (100%)	109 (100%)	73 (99%)
Cheilio inermis	232 (100%)	51 (72%)	167 (81%)	109 (100%)	74 (100%)
Lethrinus lentjan	151 (65%)	71 (100%)	152 (74%)	64 (59%)	56 (76%)
Lethrinus mahsena	151 (65%)	71 (100%)	152 (74%)	64 (59%)	56 (76%)
Lethrinus harak	151 (65%)	51 (72%)	112 (54%)	64 (59%)	56 (76%)
Calotomus carolinus	184 (79%)	22 (31%)	121 (59%)	81 (74%)	57 (77%)
Leptoscarus vaigiensis	184 (79%)	22 (31%)	121 (59%)	81 (74%)	57 (77%)
Acanthuru striostegus	181 (78%)	22 (31%)	120 (58%)	81 (74%)	56 (76%)
Cheilinus trilobatus	129 (56%	49 (69%)	141 (68%)	73 (67%)	35 (47%)
Ctenochaetus striatus	184 (79%)	2 (3%)	81 (39%)	81 (74%)	57 (77%)
Lethrinus nebulosus	48 (21%)	69 (97%)	126 (61%)	28 (26%)	17 (23%)
Parupeneus barberinus	103 (44%)	22 (31%)	66 (32%)	36 (33%)	39 (53%)
Halichoeres hortulanus	48 (21%)	49 (69%)	86 (42%)	28 (26%)	17 (23%)
Lethrinus olivaceus	48 (21%)	49 (69%)	86 (42%)	28 (26%)	17 (23%)
Lutjanus bohar	48 (21%)	49 (69%)	86 (42%)	28 (26%)	17 (23%)
Thalassoma hebraicum	48 (21%)	49 (69%)	86 (42%)	28 (26%)	17 (23%)
Hemiramphus far	103 (44%)	2 (3%)	26 (13%)	36 (33%)	39 (53%)
Sphyraena barracuda	103 (44%)	2 (3%)	26 (13%)	36 (33%)	39 (53%)
Gerres oyena	100 (43%)	2 (3%)	25 (12%)	36 (33%)	38 (51%)
Scomberoides commersonianus	100 (43%)	2 (3%)	25 (12%)	36 (33%)	38 (51%)
Plectorhinchus flavomaculatus	81 (35%)		55 (27%)	45 (41%)	18 (24%)
Acanthurus nigrofuscus		20 (28%)	40 (19%)		
Acanthurus tennenti		20 (28%)	40 (19%)		
Parupeneus indicus		20 (28%)	40 (19%)		
Scarus ghobban		20 (28%)	40 (19%)		
Siganus stellatus		20 (28%)	40 (19%)		
Zanclus cornutus		20 (28%)	40 (19%)		
Plectorhinchus gaterinus	3 (1%)		1 (1%)		1 (1%)
Rastrelliger kanagurta	3 (1%)		1 (1%)		1 (1%)
Sardinella gibbosa	3 (1%)		1 (1%)		1 (1%)
Scarus atrilunula	3 (1%)		1 (1%)		1 (1%)
Sphyraena flavicauda	3 (1%)		1 (1%)		1 (1%)
Strongylura incisa	3 (1%)		1 (1%)		1 (1%)
Abudefduf vaigiensis	3 (1%)		1 (1%)		1 (1%)

Supplementary Table 5. Full exponential random graph model results for each site. Terms in parentheses represent parameter codes in MPNet[10].

Effect	Lambda	Parameter	S.E.	t-ratio	SACF
**Site A**					
Density (EdgeA)	2.000	-7.841	0.356	-0.012	0.164
Centralization (ASA)	2.000	0.004	0.109	0.010	0.174
Closure (ATA)	2.500	0.683	0.097	0.031	0.176
Leader activity	2.000	0.817	0.181	0.052	0.085
Landing site 2 activity	2.000	0.436	0.132	-0.046	0.075
Landing site 7 activity	2.000	1.155	0.232	-0.017	-0.028
Landing site 15 activity	2.000	0.385	0.065	-0.034	0.157
Landing site homophily	2.000	3.140	0.225	-0.004	0.165
S-E triangle (Triangle XAX)	2.000	0.067	0.016	-0.009	0.174
**Site B**					
Density (EdgeA)	2.000	-6.379	0.882	-0.025	-0.040
Centralization (ASA)	2.000	0.196	0.206	-0.017	-0.029
Closure (ATA)	2.000	0.443	0.127	0.032	-0.009
Leader activity	2.000	0.977	0.188	0.035	-0.006
Landing site homophily	2.000	1.175	0.621	-0.024	-0.030
S-E triangle (Triangle XAX)	2.000	0.080	0.027	-0.011	-0.050
**Site C**					
Density (EdgeA)	2.000	-7.599	0.294	0.056	0.196
Centralization (ASA)	2.500	0.290	0.094	0.062	0.246
Closure (ATA)	2.500	0.627	0.100	0.052	0.273
Leader activity	2.000	0.839	0.160	0.083	0.162
Landing site 3 activity	2.000	0.674	0.116	0.041	0.100
Landing site 6 activity	2.000	0.275	0.089	0.063	0.264
Landing site 18 activity	2.000	0.375	0.096	-0.018	0.017
Landing site homophily	2.000	1.730	0.154	0.031	0.180
S-E triangle (Triangle XAX)	2.000	0.082	0.017	0.050	0.209
Density (EdgeA)	2.000	-7.599	0.294	0.056	0.196
**Site D**					
Density (EdgeA)	2.000	-7.307	0.590	0.045	0.066
Centralization (ASA)	2.000	0.128	0.188	0.041	0.054
Closure (ATA)	2.000	0.605	0.194	0.065	0.033
Leader activity	2.000	1.673	0.403	0.052	-0.003
Landing site 10 activity	2.000	0.826	0.307	0.074	-0.012
Landing site homophily	2.000	2.612	0.400	0.048	0.069
S-E triangle (Triangle XAX)	2.000	0.059	0.032	0.029	0.060
**Site E**					
Density (EdgeA)	2.000	-6.062	0.706	0.000	0.466
Centralization (2Star)	2.000	0.069	0.011	0.025	0.563
Centralization (ASA)	2.000	-0.296	0.221	0.002	0.490
Closure (ATA)	2.000	0.450	0.170	0.015	0.526
Leader activity	2.000	1.452	0.310	0.011	0.466
Landing site 8 activity	2.000	0.592	0.179	0.039	0.183
Landing site 16 activity	2.000	0.382	0.137	-0.026	0.261
Landing site homophily	2.000	2.501	0.350	-0.010	0.327
S-E triangle (Triangle XAX)	2.000	0.044	0.030	-0.019	0.422

Supplementary Table 6. Residual effects not well captured by our final models.

Site	Graph Statistic	Obs.	Mean	t-ratio
A	4StarA	4320	964	3.07
	5StarA	12017	1351	3.76
	Skew degreeA	3.78	2.31	4.53
C	4StarA	17546	4424	2.92
	5StarA	69885	9992	4.65
	Skew degreeA	4.69	2.92	5.08

Supplementary Table 7. Ecological, biophysical, environmental, and human impact data sources.

	Site				
	A	B	C	D	E
Biomass and Functional Richness					
source	transects	transects	transects	transects	transects
year(s)	2014	2014	2011, 2012, 2014, 2015	2012, 2014	2014, 2015
n	2	2	2, 2, 1, 1	2, 2	2, 2
Coral Cover and Rugosity					
source	NA	transects	transects	transects	transects
year(s)	NA	2016	2009	2010, 2014	2014, 2015
n	NA	17	9	9, 12	12, 12
SST					
source	6 yr average based on NOAA Coral Reef Watch 5 km dataset (https://coralreefwatch.noaa.gov/satellite/index.php)				
year(s)	2010 - 2015				
NPP					
source/method	11 yr average based on Yeager et al. (2017) [https://shiny.sesync.org/apps/msec/]				
year(s)	2002 - 2013				
Human gravity					
source/method	Total gravity of human impacts following Cinner et al. (2018). Applied to coral reefs, the gravity concept captures interactions between people and reefs as a function of the population of a place divided by the squared time it takes to travel to the reefs (travel time[16]). Total gravity accounts for the cumulative human gravity of all populated places within a 500-km radius of each reef site.				
year	2014	2014	2014	2014	2014
Fishing pressure					
source/method	Total fishing area (km2) divided by the estimated number of fishers. Estimates were obtained from key informants prior to undertaking the social data collection and were updated in the field (see SI text 'Social Networks')				

Supplementary Table 8. Sensitivity analyses. Examinations of (1) ecological conditions and biophysical, environmental, and human impact characteristics across sites with and without social-ecological network closure with site A removed; (2) ecological conditions in sites with and without social-ecological network closure in 2014; and (3) ecological conditions in sites with and without social-ecological network closure in 2014 with site A removed.

	Year	With s-e closure			Without s-e closure			Two-sample t-test	Effect size
		n	*mean*	*sd*	*n*	*mean*	*sd*	*t*(*df*)=*t*-value, *p*-value	*Cohen's D* [90% CI]
All data, Site A Removed		Sites B-C			Sites D-E				
Fishable biomass	2010-2015	8	146.33	80.47	8	64	38.35	*t*(14)=2.61, 0.01*	1.31 [0.37, 2.20]
Functional richness	2010-2015	8	0.21	0.09	8	0.08	0.06	*t*(14)=3.31, <0.01*	1.66 [0.66, 2.60]
SST	2010-2015	12	27.37	0.13	12	27.26	0.13	*t*(22)=2.01, 0.06	0.82 [0.11, 1.51]
NPP	2002-2013	2	1045.61	101.86	2	951.77	0	*t*(1)=1.30, 0.42[a]	1.30 [-0.96, 3.22][a]
Coral Cover	2009-2016	26	29.98	14.69	45	32.68	9.30	*t*(36.8)= -0.84, 0.41[a]	-0.23 [-0.64, 0.18][a]
Rugosity	2009-2016	26	1.22	0.07	45	1.22	0.08	*t*(69)= -0.03, 0.98	-0.01 [-0.41, 0.40]
Human gravity	2014	2	2658	3334.72	2	4471.5	5609.48	*t*(2)=-0.39, 0.73	-0.39 [-2.02, 1.32]
Fishing pressure	2015	2	65.5	48.79	2	153.5	21.92	*t*(2)=-2.33, 0.15	-2.33 [-4.59, 0.24]
2014		Sites A-C			Sites D-E				
Fishable biomass	2014	5	379.98	317.28	4	76.06	53.78	*t*(4.29)=2.10, 0.05*[,a]	1.25 [-0.10, 2.49][a]
Functional richness	2014	5	0.37	0.13	4	0.08	0.08	*t*(7)=3.95, <0.01*	2.65 [0.10, 4.18]
2014, Site A removed		Sites B-C			Sites D-E				
Fishable biomass	2014	3	231.09	68.80	4	76.06	53.78	*t*(5)=3.37, 0.01*	2.57 [0.68, 4.30]
Functional richness	2014	3	0.29	0.06	4	0.08	0.08	*t*(5)=3.63, <0.01*	2.78 [0.81, 4.57]

[a]Satterthwaite's formula[17] was used to approximate the degrees of freedom to account for unequal variance.

.

Supplementary References

1 McClanahan, T., Muthiga, N. A. & Abunge, C. A. Establishment of community managed fisheries' closures in Kenya: early evolution of the tengefu movement. *Coastal Management* **44**, 1-20 (2016).

2 Froese, R. & Pauly, D. *FishBase*, www.fishbase.org (2019).

3 Hobson, E. S. Feeding relationships of teleostean fishes on coral reefs in Kona, Hawaii. *Fish. Bull.* **72**, 915-1031 (1974).

4 Lusher, D., Koskinen, J. & Robins, G. *Exponential random graph models for social networks: Theory, methods, and applications*. (Cambridge University Press, 2012).

5 Wang, P., Robins, G., Pattison, P. & Lazega, E. Exponential random graph models for multilevel networks. *Social Networks* **35**, 96-115 (2013).

6 Robins, G., Elliott, P. & Pattison, P. Network models for social selection processes. *Social Networks* **23**, 1-30 (2001).

7 Wang, P., Robins, G. & Matous, P. in *Multilevel Network Analysis for the Social Sciences* Vol. 12 *Methodos Series* (eds Emmanuel Lazega & Tom A. B. Snijders) Ch. 6, 125-143 (Springer International Publishing, 2016).

8 Alexander, S. M., Barnes, M. L. & Bodin, Ö. Untangling the drivers of community cohesion in small-scale fisheries. *International Journal of the Commons* **12** (2018).

9 Lubell, M., Robins, G. & Wang, P. Network structure and institutional complexity in an ecology of water management games. *Ecology and Society* **19** (2014).

10 PNet: program for the simulation and estimation of exponential random graph models (Melbourne School of Psychological Sciences, The University of Melbourne, 2009).

11 Snijders, T. A. Markov chain Monte Carlo estimation of exponential random graph models. *Journal of Social Structure* **3**, 1-40 (2002).

12 McClanahan, T. & Kosgei, J. Redistribution of benefits but not defection in a fisheries bycatch-reduction management initiative. *Conservation Biology* (2017).

13 Mouillot, D., Graham, N. A., Villéger, S., Mason, N. W. & Bellwood, D. R. A functional approach reveals community responses to disturbances. *Trends in Ecology & Evolution* **28**, 167-177 (2013).

14 McClanahan, T. R. Kenyan coral reef lagoon fish: effects of fishing, substrate complexity, and sea urchins. *Coral Reefs* **13**, 231-241 (1994).

15 Cinner, J. E. *et al.* Gravity of human impacts mediates coral reef conservation gains. *Proceedings of the National Academy of Sciences*, 201708001 (2018).

16 Maire, E. *et al.* How accessible are coral reefs to people? A global assessment based on travel time. *Ecology Letters* **19**, 351-360 (2016).

17 Satterthwaite, F. E. An approximate distribution of estimates of variance components. *Biometrics Bulletin* **2**, 110-114 (1946).
